# ROP18 Is a Rhoptry Kinase Controlling the Intracellular Proliferation of Toxoplasma gondii

**DOI:** 10.1371/journal.ppat.0030014

**Published:** 2007-02-16

**Authors:** Hiba El Hajj, Maryse Lebrun, Stefan T Arold, Henri Vial, Gilles Labesse, Jean François Dubremetz

**Affiliations:** 1 UMR 5539, Centre National de la Recherche Scientifique, Université de Montpellier 2, Montpellier, France; 2 Centre de Biochimie Structurale, Centre National de la Recherche Scientifique, Université Montpellier 1, Montpellier, France; 3 Centre de Biochimie Structurale, Institut National de la Santé et de la Recherche Médicale, Montpellier, France; Northwestern University Medical School, United States of America

## Abstract

Toxoplasma gondii is an obligate intracellular parasite for which the discharge of apical organelles named rhoptries is a key event in host cell invasion. Among rhoptry proteins, ROP2, which is the prototype of a large protein family, is translocated in the parasitophorous vacuole membrane during invasion. The ROP2 family members are related to protein-kinases, but only some of them are predicted to be catalytically active, and none of the latter has been characterized so far. We show here that ROP18, a member of the ROP2 family, is located in the rhoptries and re-localises at the parasitophorous vacuole membrane during invasion. We demonstrate that a recombinant ROP18 catalytic domain (amino acids 243–539) possesses a protein-kinase activity and phosphorylate parasitic substrates, especially a 70-kDa protein of tachyzoites. Furthermore, we show that overexpression of ROP18 in transgenic parasites causes a dramatic increase in intra-vacuolar parasite multiplication rate, which is correlated with kinase activity. Therefore, we demonstrate, to our knowledge for the first time, that rhoptries can discharge active protein-kinases upon host cell invasion, which can exert a long-lasting effect on intracellular parasite development and virulence.

## Author Summary

Apicomplexa are unicellular eukaryotes that cause a number of diseases, including malaria. Most of them are obligate intracellular parasites, developing in a parasitophorous vacuole (PV) within their host cell. PV formation during invasion is associated with the exocytosis of parasite secretory organelles named rhoptries, whose role is unknown. Toxoplasma gondii is a model Apicomplexa responsible for toxoplasmosis, a fatal congenital or opportunistic infection in humans and animals. We have studied a novel rhoptry protein dubbed ROP18, which is translocated to the PV membrane upon invasion. ROP18 belongs to a family of rhoptry proteins that share homologies with serine-threonine kinases, but those described so far lack residues critical for enzyme activity. We show that ROP18 possesses all the features needed to be active, and we experimentally demonstrate this activity, which phosphorylates at least one parasite protein. We show that overexpression of ROP18 causes a dramatic increase in parasite multiplication rate that is correlated with kinase activity, and likely dependent on a PV membrane modification. We therefore demonstrate that rhoptries can discharge active protein-kinases upon invasion, which can exert a long-lasting effect on intracellular parasite development and virulence.

## Introduction

Toxoplasma gondii is an obligate intracellular parasite belonging to the protozoan phylum Apicomplexa, which includes a large number of human and animal parasites responsible for diseases such as malaria, toxoplasmosis, coccidiosis, and cryptosporidiosis. As for all other members of the phylum, host cell invasion by T. gondii involves specialized apical organelles of the invasive stage, namely micronemes and rhoptries, which discharge their contents successively [1,2]. The exocytosis of micronemal proteins is associated with gliding and attachment to the host cell [3–6]. Then, a complex of microneme and rhoptry neck proteins forms a moving junction with the host cell plasma membrane that propels the parasite within the developing parasitophorous vacuole [7,8]. Subsequently, proteins of the bulb of the rhoptries (ROP proteins) become associated with the parasitophorous vacuole membrane (PVM) that forms from host plasma membrane and rhoptry components during invasion [9]. Among rhoptry proteins is a series of related proteins, the ROP2 family [10–12], named after the ROP2 protein, which is translocated into the PVM during invasion [13]. The N-terminal (Nt) domain of ROP2 has been shown to interact with the mitochondrial import machinery and to mediate the association of host mitochondria to the PVM [14]. Targeted depletion of ROP2 using a ribozyme-modified antisense RNA strategy results in disruption of rhoptry biogenesis and affects cytokinesis, association of host cell mitochondria with the PVM, host cell invasion, and virulence in mice [15]. Several other members of the family have been characterized more recently, and they are also targeted to the PVM upon invasion [16–18].

The importance of ROP2 and the fact that the parasite is synthesizing simultaneously several ROP2 homolog proteins suggest that these proteins serve crucial functions; yet, the apparent indispensability of ROP2 suggests that they may not complement one another and may have distinct functions. We have recently shown that the ROP2 family could be expanded to at least 12 members, some of which show a full set of features compatible with protein-kinase activity, whereas ROP2 and its closest relatives have lost some of these features [12]. This raises the question of the role played by these proteins. Indeed, parasitic kinase(s) acting on host cell inhibitor of nuclear factor κB (IκB) have been suggested to be present at the PVM level [19]. Thus, T. gondii could be capable of manipulating the host cell machinery using its own kinases to favour its survival and development.

Recently, many investigations have focused on searching protein-kinases in unicellular parasites, based on the fact that the vast phylogenetic distance between the organisms and their vertebrate hosts may have generated divergences in the properties of their protein-kinases that could be exploited for specific inhibition of the parasite enzymes [20–23].

This has prompted us to study the new members of the ROP2 family predicted to possess a fully functional protein-kinase domain. We report here the cloning and characterization of ROP18, a novel ROP2-related rhoptry protein that is translocated to the PVM during invasion. We show that ROP18 is a protein-kinase that can phosphorylate a tachyzoite substrate of 70 kDa. We therefore demonstrate that rhoptries can discharge active kinases at the parasite–host cell interface upon cell invasion. In addition, overexpression of ROP18 in tachyzoites led to a dramatic stimulating effect on intracellular parasite multiplication, strongly suggesting that this protein plays a role in the control of parasite proliferation and may therefore be involved in T. gondii virulence.

## Results

### Analysis of ROP18 Sequence

**Figure 1:**
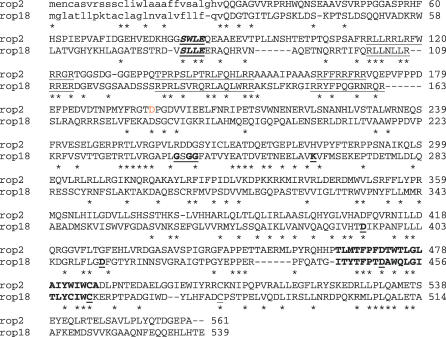
Alignment of the ROP2 and ROP18 Sequences The identities are underlined by asterisks. The predicted signal sequences are in lower case. The three arginine-rich regions in the Nt of the proteins are underlined. The SΦXE motif, putative maturation site of the pro-region, is in bold italics and underlined; the kinase activity key amino acids are in boldface and underlined (G stretch, catalytic lysine, and key aspartates); the hydrophobic stretch predicted as transmembrane is in bold letters; and the two conserved cysteins in the C-terminal domain are underlined.

The open reading frame (ORF) included in the expressed sequence tag (EST) Cluster 100121072 (APIDBest, http://www.apidb.org/apidb) corresponding to the ROP18 protein has been amplified from T. gondii genomic DNA and sequenced [12]. The ROP18 protein–deduced primary sequence aligns with the ROP2 sequence with 25% identity (Figure 1). ROP18 is more closely related to ROP5 (28%) than to the other ROP2 prototypes, such as ROP2, 4, 7, and 8.

As other ROP2 family proteins, ROP18 contains an Nt peptide signal sequence, with a predicted cleavage site between residues 28 and 29 (the second Met of the ORF has been considered as the start codon by homology to ROP2, and used as position 1) or between residues 32 and 33, according to SignalP.

Almost all rhoptry proteins described so far in *T. gondii,* including members of the ROP2 family proteins (ROP2, 4, and 7), are synthesized as pro-proteins that are subjected to proteolytic cleavage during trafficking to rhoptries removing the Nt pro-region [10,17,24]. The exact site of cleavage for the ROP2 family proteins is unknown, but the sequence (SWLE) present at the end of the pro-domain of ROP2, ROP4, and ROP8 has been suspected to be the cleavage site by the maturase TgSUB2 (the proposed consensus being SΦXE, where Φ represents bulky hydrophobic residues and X is any amino acid [25]). ROP5 lacks this sequence and is not processed [18]. ROP18 contains at the same location the sequence SLLE, and may therefore be cleaved after amino acid 82.

Following the predicted cleavage site, several arginine-rich stretches are observed in ROP2 family proteins, including ROP2, 4, 5, 7, and 8 [12]. Two such arginine-rich segments are clearly recognized in ROP18 sequence at positions 101–113 and 129–142, and a third one at 152–163 is more degenerate. However, the precise role of these basic and amphipathic segments remains unknown, although they may serve to anchor proteins onto membrane surfaces. These stretches are followed by a linker region (residues 173–233) whose function and structure are unknown.

A putative serine/threonine protein-kinase domain in the C-terminal (Ct) half of the ROP18 sequence was identified by PSI-BLAST search [26] with significant e-value (below e^−7^). However, this domain comprises a hydrophobic stretch conserved in other ROP2 family proteins that has been previously considered as a transmembrane segment. The corresponding segment in ROP18 is weakly predicted by TopPred as a putative transmembrane segment (443 and 463) with a score of 0.614. Our recent sequence analysis of all ROP2 family sequences has led us to rule out the transmembrane prediction for this conserved segment [12]. We rather predicted that the Ct region (234–539 in ROP2) adopts a protein-kinase fold. In order to confirm this hypothesis, molecular modelling was performed on ROP18.

### Structural Characterization of ROP18

**Figure 2:**
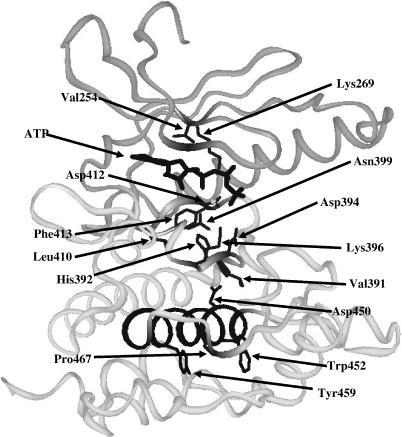
Structural Conservation and Protein-Kinase Fold in ROP18 and Related Proteins The side chains of residues strictly conserved among PDB1PHK and various ROP2s (ROP17, 18, 2L3, and 2L4) are shown in black and wire-frame on the backbone of PDB1PHK. The hydrophobic segment (corresponding here to residues 443 to 463 in ROP18) is in black colour and appears buried into the all-alpha domain. The ATP molecule is shown in black and thick wire-frame in order to localise the active site in which conservation corresponds mainly to catalytic residues. Residue numbering refers to the ROP18 sequence.

Comparative modelling was initiated using fold-recognition through the meta-server @TOME [27]. Significant scores of fold-compatibility were obtained with various serine/threonine kinases (see results at http://www.infobiosud.cnrs.fr/bioserver/ROP/suppl.html) despite a low overall sequence identity (∼20% over the whole Ct domain). Molecular modelling of this domain (Figure 2) further demonstrated the conservation of the protein-kinase fold, especially all the residues critical for the domain stability and the protein-kinase activity [28]. The hydrophobic segment appears to be completely buried inside the helical domain of the protein core and to bear residues essential for protein stability (including D450, W452, and G455). Indeed, it corresponds to the Hanks motif “DxxxxG” numbered as IX. Among the other conserved motifs, those involved in catalysis, regulation, and peptide recognition were further scrutinized using the theoretical models. Motifs I, II, VIb, VII, and VIII as defined by Hanks [28] were clearly detected. The catalytic lysine (K266 in ROP18) and aspartate (D394) residues were present. The region 427 to 435 perfectly matched the Hanks motif VIII of serine/threonine kinases. ROP18 is unique among ROP2-like proteins in having this peptide-binding motif perfectly conserved. The sub-sequence GTP (427-GTP-429 in ROP18) is expected to recognize serine or threonine residues to be phosphorylated. This functional prediction was confirmed by the sequence of the motif VIb (392-HTDIKPAN-399 in ROP18) [29]. This motif bears a consensus sub-sequence, “KpeN,” that is specific to serine/threonine kinases (versus aarN in tyrosine kinases). The absence of an arginine at the second residue position of this motif in ROP18 (bearing a threonine T393 instead) suggests that ROP18 does not need phosphorylation of its activation loop to become active.

These in silico predictions were further supported by experimental data gained on another member of the ROP2 protein family sharing the same predicted structural features [12]. Indeed, refolded recombinant ROP18 was rather unstable and could not be obtained in sufficient amounts, whereas previous work had shown that ROP2 could be obtained directly as a stable soluble recombinant protein [30]. Dynamic light scattering (DLS) spectra of both recombinant proteins confirmed their size similarity in solution (Figure S5).

The existence of a soluble and compact domain was evaluated by small angle X-ray scattering (SAXS) experiments performed on a recombinant ROP2 (196–561) construct at high protein concentration (up to 18 mg/ml). SAXS data on ROP2 were recorded to a maximum resolution of s = 4.63 nm^−1^ (Figure S2). Low resolution data showed only minor signs of aggregations, and the Guinier plot followed a straight line between s*Rg limits of 0.87 and 1.25 (Rg being the radius of gyration). The Rg calculated from the Guinier plot (3.90 nm) was in very good agreement with the one obtained by GNOM (3.89 nm). GNOM analysis also indicated the maximum particle diameter to be 13.5 nm. By comparison with the I0 value of a BSA standard, a molecular weight of 37 kDa was estimated for recombinant ROP2, in reasonable agreement with a calculated molecular weight of 42 kDa for a monomer. Ab initio shape calculations yielded two-lobed 40 × 40 × 65 Å ellipsoidal structures with an ∼75 Å tail, a form which is already apparent from the distance distribution (Figure S3). The ellipsoidal structure compares very well in shape and size with a typical protein-kinase domain (Figure S4). SAXS and homology modelling data suggest that, going from Ct to Nt, the 40 N-terminal residues additional to the protein-kinase domain first run over one side of the C-terminal lobe (up to residue 40–35), and then form an unstructured and solvent-exposed extension. Trp39 might be important for pinning the Nt to the C-terminal lobe. The scattering curve calculated from a homology model of ROP2 fits the experimental SAXS data well, given the only modest sequence homology (Figure S2). Altogether, SAXS data corroborate that recombinant ROP2 consists of a protein-kinase domain with an unstructured 40 residue–N-terminal tail. The size observed for freshly refolded ROP18 by DLS (see below) and the significant sequence similarity shared by ROP18 and ROP2 suggests that these two proteins (as well as other ROP2-like proteins) possess the same structural organization containing a folded protein-kinase domain. Similarly, ultraviolet circular dichroism (UV-CD) measurement (wavelength 195–260 nm; unpublished data) suggested that both ROP18 and ROP2 proteins are mainly composed of alpha-helices in agreement with their predicted fold. In conclusion, ROP18 is predicted to be composed of an N-terminal domain of unknown structure, while the large C-terminal domain would be folded as a soluble and functional serine/threonine protein-kinase.

### The ROP18 Gene Encodes a Rhoptry Protein That Is Proteolytically Processed in the Secretory Pathway

**Figure 3:**
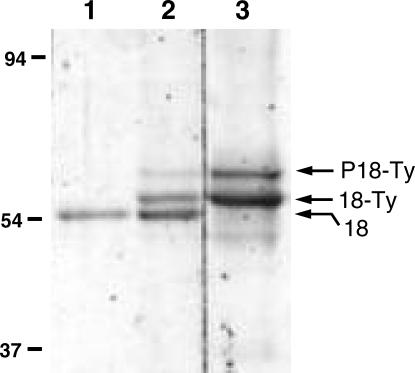
Western Blot Analysis of ROP 18 and ROP18-Ty Western blot of HX (lane 1) and of ROP18-Ty–transfected tachyzoites (lanes 2 and 3) were probed with rabbit anti-ROP18 affinity-purified antibodies (lanes 1 and 2) or mAb anti-Ty (lane 3). Native ROP18 migrates at 55 kDa (18), whereas ROP18-Ty is at about 56 (18-Ty) and its unprocessed precursor at about 60 kDa (P18-Ty). No unprocessed form of native ROP18 is detected.

To investigate the expression and subcellular localization of ROP18, we raised a polyclonal serum against a recombinant ROP18 protein. On immunoblots of tachyzoites, anti-ROP18 antibodies reacted with a single protein with an apparent molecular mass of 55 kDa (Figure 3). The specificity of the antibodies was assessed by Western blotting transgenic parasites expressing a Ty-tagged version of ROP18. When ROP18-Ty tachyzoites were analyzed, two bands at 56 and 60 kDa were found with anti-Ty monoclonal antibody (mAb) (Figure 3). The same bands were detected when probing with anti-ROP18, together with the 55-kDa band, confirming the antibody specificity. The mobility shift observed for the 56-kDa band is consistent with the addition of a Ty-1 epitope. The 60-kDa band was interpreted as unprocessed ROP18-Ty protein (see below).

**Figure 4:**
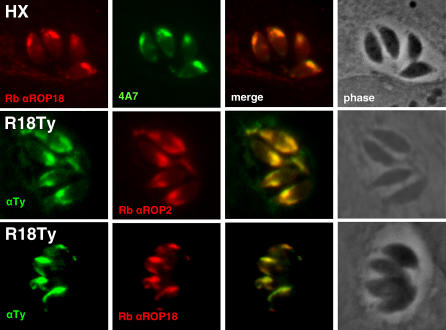
IFA Localisation of ROP18 in Intracellular Parasites In the upper row, intracellular HX tachyzoites were reacted with pre-immune or immune rabbit anti-ROP18 antibodies combined with mAb anti–ROP2-ROP4. In the lower rows, intracellular ROP18-Ty–transfected tachyzoites were reacted with mAb anti-Ty, combined with either rabbit anti-ROP2 serum or rabbit anti-ROP18 antibodies.

When analyzed by immunofluorescence assay (IFA) on T. gondii–infected human foreskin fibroblasts (HFFs), ROP18 was found at the apical end of RHΔhx (HX) tachyzoites, co-localizing exactly with ROP1 (Figure 4, HX). In transgenic parasites expressing ROP18-Ty, the anti-Ty antibodies labelled the rhoptries (Figure 4, R18Ty), and, in some cases, vesicles located between the rhoptries and the nucleus (not shown).

**Figure 5:**
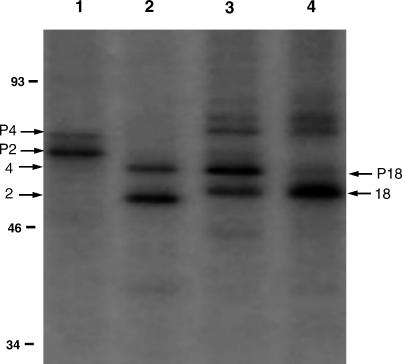
Pulse-Chase Analysis of ROP18 Biosynthesis HFFs were infected with the ROP18-Ty–transfected tachyzoites for 24 h, then labelled for 20 min with [^35^S] methionine/cysteine and either harvested (lanes 1 and 3) or chased for 1 h (lanes 2 and 4). Then, the NP40 lysate was immunoprecipitated with mAb anti–ROP2–4 (T_3_ 4A_7_, lanes 1 and 2) or mAb anti-Ty (lanes 3 and 4). Lane 3 shows a major band at 60 kDa (P18), whereas a 56-kDa major protein (18) is found in lane 4. The control with mAb T_3_ 4A_7_ that immunoprecipitates ROP2 (2) and ROP4 (4) shows parallel processing of both proteins from higher molecular mass precursors (P4 and P2).

To determine whether ROP18 is processed during trafficking to rhoptries, we studied the biosynthesis of ROP18 by pulse-chase metabolic labelling with [^35^S] methionine. For this analysis, the transfected strain ROP18-Ty was used. After a 20-min pulse, one major labelled protein of 60 kDa and a minor of 56 kDa were immunoprecipitated by mAb anti-Ty (Figure 5). The 56 kDa was strongly enriched when a 1-h chase was performed, suggesting that ROP18 is processed in the biosynthetic pathway. When compared with ROP2-ROP4 immunoprecipitated on mAb T_3_ 4A_7_, the mature 56-kDa form of ROP18-Ty appeared slightly earlier than mature ROP2-ROP4. Whether this is due to the expression of ROP18-Ty under a tubulin promoter rather than under its native one, or to different kinetics of trafficking in the pathway, remains to be established. The persistence of unprocessed ROP18-Ty observed on Western blots (Figure 3) could also be explained by some untimely synthesis due to the tubulin promoter that may lead to accumulation in vesicles located between the rhoptries and the nucleus, or trafficking to compartments that do not contain the processing enzymes when rhoptries are not being produced.

This demonstration of proteolytic processing, together with the presence of the SLLE motif at the expected cleavage site in the sequence, tends to reinforce the hypothesis of TgSUB2 being the processing enzyme [25], as the only unprocessed member of the family known so far is ROP5, which lacks this motif.

### ROP18 Is Secreted during Host Cell Invasion and Associates with the PVM

**Figure 6:**
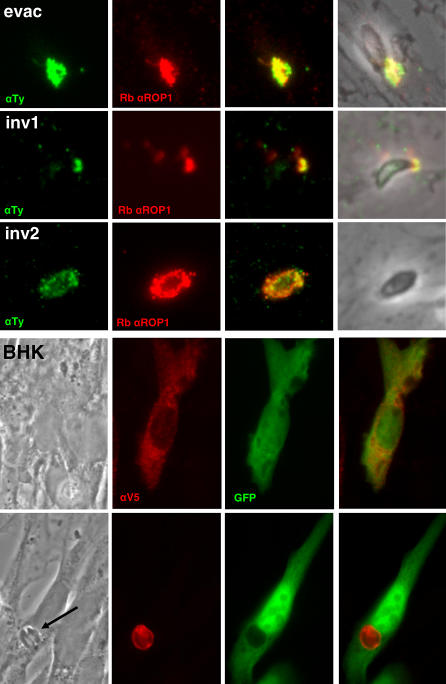
ROP18 Is Secreted during Host Cell Invasion and Associates with the PVM The upper panel shows evacuoles of a Cyt-D–arrested ROP18Ty parasite (evac), of an invading tachyzoite (inv1), and of a newly invaded tachyzoite (inv2) of the same clone, all reacted with mAb anti-Ty and rabbit anti-ROP1 antibodies. In all cases, the ROP1 and Ty labels co-localise, demonstrating the translocation of ROP18Ty in the evacuoles or in the PVM. The lower panel shows two BHK cells that have been transfected with Plasmid pTracer-ROP18ΔPro, one of which was infected later by T. gondii (arrow). Both cells express GFP in the cytosol; in the non-infected cell, the V5 epitope is detected as dots scattered in all cytosol, whereas in the infected cell, it is restricted to the PVM, showing a strong tropism of ROP18 for this membrane.

We then investigated the fate of ROP18 during HFF invasion. Rhoptries are discharged during the invasion process [2,9,13], and their contents associate with the nascent vacuole membrane. When invasion is interrupted with cytochalasin-D (Cyt-D), rhoptry-derived vesicles named evacuoles accumulate in the host cell cytoplasm [31]. When Cyt-D–arrested parasites were labelled with anti-ROP18, we found that ROP18 was associated with evacuoles (Figure 6, evac). ROP18 was also associated with the PVM of invaders and of recently invaded parasites, co-localizing with ROP1 (Figure 6, inv1 and inv2).

The tropism of ROP18 for the PVM was also confirmed upon infection of BHK21 cells that transiently expressed ROP18 (Figure 6, BHK). The nucleotide coding sequence corresponding to mature ROP18 (ROP18ΔPro, amino acids 83–539, by deletion of the peptide signal and putative propeptide) was cloned in frame with a sequence coding for the V5 epitope, in the mammalian expression vector pTracer-A, which allows co-expression of the sequence of interest and of the green fluorescent protein (GFP). GFP is expressed in the cytoplasm and its intrinsic fluorescence allows direct visualization of transfected cells. In these cells expressing ROP18ΔPro, anti-V5 antibodies produced a punctuate labelling homogeneously distributed in the cytosol (Figure 6, BHK, upper row). When these cells were infected with tachyzoites 4 h after transfection and fixed 16 h after infection, the anti-V5 labelling was found prominently around the PVM, with some extension in the parasitophorous vacuole that may correspond to the PVM-derived intra-vacuolar network (Figure 6, BHK, lower row; Figure S1). In contrast, the distribution of GFP was unchanged. Control PVM in non-transfected cells (GFP-negative cells) were not labelled (not shown). These results indicate that ROP18 possesses a strong affinity for the PVM.

Collectively, these results demonstrate that ROP18 is a rhoptry protein secreted during the invasion process that associates with the PVM-surrounding intracellular parasites.

### ROP18 Is an Active Kinase

**Figure 7:**
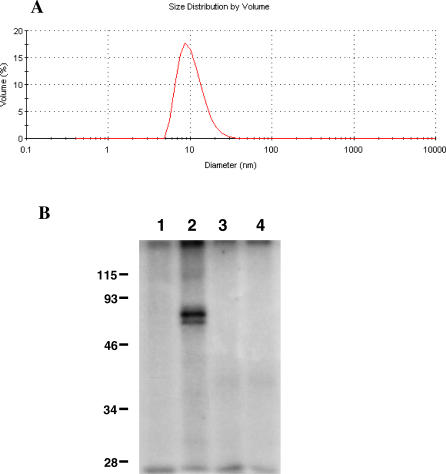
ROP18 Is an Active Kinase (A) DLS of recombinant kinase domain of ROP18. Refolding was performed on the recombinant ROP18 solubilized in Gdn-HCl and monitored by DLS. Ten-fold dilution was directly performed into the cuvette and light scattering measured after 1 min. Trial-and-error led to an optimal solution in which refolded ROP18 appeared as a monodisperse entity roughly 10 nm in size (without taking into account the viscosity of the refolding buffer). (B) SDS-PAGE autoradiography of a kinase assay of the recombinant kinase domain of ROP18. In lanes 2 and 4, the recombinant His-tagged ROP18 produced in E. coli was refolded, purified, and incubated with tachyzoite lysate (lane 2) or HFF lysate (lane 4) in the presence of [γ^32^P-ATP], whereas in lanes 1 and 3, the recombinant His-tagged ROP18 mutated on aspartate 394 was used with tachyzoites (lane 1) or HFF lysate (lane 3).

Since ROP18 showed a full set of features compatible with protein-kinase activity, we investigated the predicted catalytic properties of this protein. We therefore expressed the catalytic domain with a Ct His-tag in Escherichia coli. The recombinant protein was found in bacterial inclusion bodies in the various conditions tested so far (unpublished data). We therefore used a denaturation-refolding procedure to purify the recombinant protein. Refolding was monitored by light scattering (Figure 7A) and was confirmed by tryptophan fluorescence (unpublished data). The refolded protein was incubated with either heat-inactivated parasite or HFF lysate and assayed for kinase activity (Figure 7B). A major phosphorylated band of 70 kDa and a minor one of 68 kDa were detected in parasite (Figure 7B, lane 2), but not in HFF lysate (Figure 7B, lane 4). Autophosphorylation by the refolded kinase was not observed (unpublished data).

To confirm that the observed phosphorylation was due to the catalytic activity of ROP18, we generated a recombinant catalytic domain with a mutation of aspartic acid D^394^ (domain VIb, according to Hanks [28]), required for the activity, to an alanine. We expressed and refolded the mutated catalytic domain as done for the native catalytic domain and compared the activity of equal amounts of both proteins. The kinase assay with the mutated protein on parasite extracts did not lead to any significant labelling (Figure 7B, lanes 1 and 3), clearly demonstrating that mutation of the ROP18 catalytic aspartic acid D^394^ to an alanine leads to the loss of kinase activity.

### Overexpression of ROP18 Leads to Increased Intracellular Parasite Proliferation

Cultivation of the transfected ROP18-Ty strain routinely showed an earlier release of parasites compared with that of wild-type. We therefore investigated whether this was due to higher invasion rate or faster intracellular multiplication. To make sure that a position effect of the transformation was not involved, we duplicated the experiments with parasite clones isolated from two independent ROP18-Ty transfections. Evaluating the invasion rate of the various parasites did not show any significant difference between wild-type and transfected clones (not shown). In contrast, when counting the number of parasites per vacuole at 16 h after infection, we observed a significant increase in parasite proliferation in the ROP18-Ty–transfected parasites, compared with that of wild-type tachyzoites. The results of five experiments led to a mean reproduction rate of 2.47 ± 0.39 parasites per vacuole at 16 h after infection for wild-type, whereas the ROP18-Ty showed a rate of 4.07 ± 0.34 (Figure S6). A statistical analysis of these data using Student's *t*-test led to a *p*-value of 0.001.

**Figure 8:**
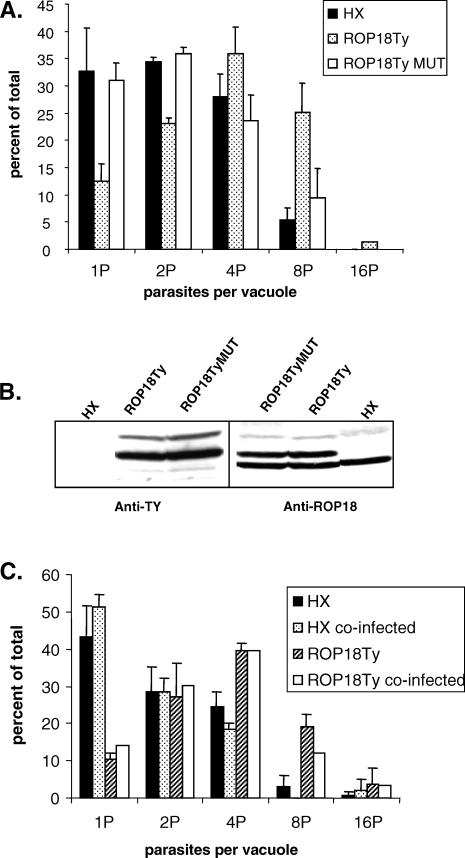
Overexpression of ROP18 Leads to Increased Intracellular Parasite Proliferation (A) Intracellular proliferation rate at 16 h post-invasion by wild-type tachyzoites (HX) and tachyzoites expressing an additional copy of ROP18-Ty (ROP18Ty) or a D^394^A-mutated version thereof (ROP18Ty MUT). This graph corresponds to one representative experiment and clearly shows a drift towards higher number of parasites per vacuole for the ROP18Ty parasites compared with that of either HX or ROP18Ty-MUT parasites. Error bars correspond to standard deviation between the triplicate coverslips. (B) Western blot of equal number of HX, ROP18Ty, and ROP18Ty MUT tachyzoites probed with anti-Ty or with anti-ROP18 showing similar expression of the Ty-tagged copy of ROP18 in both transfectants. (C) Proliferation rates of HX and ROP18Ty tachyzoites in a co-infection experiment, at 16 h post-invasion, showing that co-infection of a single cell does not modify the respective behaviour of either parasite type (HX or ROP18 are parasites alone in one cell, co-infected corresponds to vacuoles found in a cell containing also the other type, error bars correspond to standard deviation between triplicate coverslips).

In order to know whether this property was related to the enzymatic activity of ROP18, we created transfectants expressing a D^394^A-mutated ROP-18Ty. The ROP18-TyD^394^A localization was verified by IFA. As expected, the protein was present in the rhoptries and secreted during the invasion process. By counting the number of parasites per vacuoles in three experiments, we showed that the multiplication rate of ROP18-TyD^394^A–transfected parasites at 16 h (2.40 ± 0.45) was not significantly different from wild-type (Figure S6). A graph of one typical experiment showing the relative distribution of the number of parasites per vacuole at 16 h post-infection is shown in Figure 8A. The level of expression of ROP18-Ty and ROP18-TyD^394^A proteins was verified to be equivalent by quantification on Western blots of equal amounts of parasites (Figure 8B).

### The Proliferation Effect Is Vacuole-Specific

In order to evaluate whether the presence of an overexpressor in a cell could influence the reproduction rate of a co-infecting wild-type parasite, we performed a co-infection experiment with HX- and ROP18-Ty–transfected parasites. The two types of vacuoles were differentiated by IFA on the Ty-tag expression. This experiment showed that there was no influence of co-infection, with both parasite types behaving as in the single infections experiments, regardless of the presence of the other type in the same cell (Figure 8C).

Therefore, we obtained evidence that overexpression of an active ROP18 kinase leads to an increase in the rate of parasite replication, but that this effect is restricted to the vacuole containing these parasites.

## Discussion

We have identified an active kinase stored in T. gondii tachyzoites rhoptries, which is secreted in the host cell during invasion and is involved in the intracellular proliferation of the parasite. The simultaneous expression by the parasite of such a family of closely related proteins is still poorly understood. All of these proteins possess a region sharing significant homologies with the canonical kinase domain as described by Hanks [12,28]. Some of them, like ROP4, 5, 7, and 8, lack the glycine loop and the catalytic aspartic acid required for activity. Others, like ROP2, lack the glycine loop, although having kept the catalytic loop, they may still interact with a substrate without being able to phosphorylate. Remarkably, several other members of the family possess the complete set of features needed for kinase activity, which led us to investigate them further. We focused our attention on ROP18. The characteristic features of the family, such as rhoptry location, ORF size, hydrophobic segment near Ct, and arginine-rich stretches near the Nt [12], are well conserved in ROP18. We could express and refold as an active protein-kinase its complete Ct domain (243–539). We showed that it was indeed capable of phosphorylating parasite proteins. These findings demonstrate directly the presence of an active kinase in T. gondii rhoptries.

In T. gondii lysate, a major 70-kDa protein and a minor one of 68 kDa are phosphorylated; these sizes do not correspond to any parasitic protein characterized so far. Moreover, we do not know whether the negative result obtained with HFF lysates corresponds to a total absence of activity on host cell substrates, or to a defect in the experimental procedure impairing the activity.

We and others have shown that several members of the ROP2 family are translocated to the parasitophorous membrane upon invasion [13,16–18]. ROP18 follows the same route. In addition, we show that, when expressed in the cytoplasm of the host cell, ROP18 also homes to the PVM, suggesting a specific interaction with this membrane. In this location, ROP18 could modify other PVM proteins (such as another rhoptry protein or a dense granule protein) or signal and/or control host cell functions. What phosphorylation(s) occurs is yet to be identified, but two related events have already been described, namely the phosphorylation of ROP4 on several serine/threonine residues after translocation in the PVM [16], and the phosphorylation of host IκB that correlates with the activation of NF-κB, which is required for the inhibition of apoptosis [19]. As several other ROP proteins are also putative rhoptry kinases [12,32], the parasite is likely to modulate several host cell function or PVM properties soon after entry. The harnessing of the host cell by T. gondii was demonstrated by Blader et al. [33], who showed a wide range of changes in host cell transcription pattern after parasite invasion. Our observation that overexpression of ROP18 increases parasite proliferation rate, with this property being strictly linked to the protein-kinase catalytic activity, fits perfectly with these data. Such a shortening of the parasite cell cycle triggered by overexpression of a parasite protein has not been described so far. It tends to suggest that the mutants are metabolically more efficient either by activating the cell metabolism for their benefit, or by getting their supply from the host cell more efficiently. A modification of the PVM would fit with the second possibility, which is consistent with our observation that the effect of overexpression does not extend to other vacuoles in the same cell. In addition, as the length of the cell cycle differs between T. gondii strains, and as more virulent strains have higher multiplication rates, a direct connection could exist between expression of ROP18 and virulence. Such a correlation has actually been independently observed by genetic mapping of T. gondii virulence [34,35].

In conclusion, we have shown here that ROP18 is a protein-kinase belonging to the ROP2 family of rhoptry proteins. To our knowledge, we have provided the first direct demonstration of the presence of an active kinase in T. gondii rhoptries; in addition, we have shown a direct effect of the expression of this protein on the proliferation rate of the parasite, suggesting a possible role in virulence, which expands the part played by the ROP2 family proteins in the biology of *T. gondii.*

## Materials and Methods

### Host cell and parasite cultures.

All parasites were maintained by serial passage in HFFs grown in Dulbecco's modified Eagle medium (DMEM) (GibcoBRL, http://www.invitrogen.com) supplemented with 10% fetal calf serum (FCS) and 2 mM glutamine. Tachyzoites of the RH strain of T. gondii [36] and of HX deleted for hypoxanthine guanine phosphoribosyl transferase [37] were used throughout the study. BHK-21 (baby hamster kidney) cells (ATCC CCL-10) were grown in BHK-21 medium (GibcoBRL) supplemented with 5% FCS, 2 mM tryptose, 100 U/ml penicillin, and 100 μg/ml streptomycin.

Invasion and intracellular tachyzoite multiplication rates were measured on HFFs plated on 12-mm coverslips in 24-well plates and fixed 16 h after infection by equal numbers of freshly released tachyzoites. In some experiments, uninvaded parasites were washed 30 min after contact with the cells to avoid possible bias due to differences in kinetics of invasion. Coverslips were fixed and stained with eosine-methylene blue (RAL 555) and then mounted permanently (Pertex; Microm Microtech France, http://www.microm.fr). Fields were randomly selected, and the number of vacuoles per field and the number of parasites per vacuole were counted using a 40× objective in ten fields per coverslip, with three coverslips per assay. Five independent experiments were performed. Data were analyzed using Student's *t*-test. A *p*-value less than 0.05 was regarded as significant. In one experiment, coverslips were co-infected simultaneously by both wild-type and transfected parasites, and counts were performed after anti-TY IFA (see below) to differentiate between both types and compare their respective proliferation in single and double infections.

### Antibodies.

Antibodies used in this study included mAb anti-Ty-1 tag [38], mAb T_3_ 4A_7_ specific for ROP2, 3, and 4 [10], anti-recombinant ROP1 and ROP2 rabbit sera (J. F. Dubremetz and O. Mercereau-Puijalon, unpublished data), and a rabbit anti-ROP18 obtained by rabbit immunization (see below).

### Cloning procedures and plasmids construct.

Preliminary genomic and/or cDNA sequence data was accessed via ToxoDB (http://www.toxodb.org) and/or the Toxoplasma gondii Genome Project (http://www.tigr.org/tdb/t_gondii).

The ROP18 cloning was based on the EST cluster (100121072) found in ToxoDB APIDBest (http://www.apidb.org/apidb). The ROP18 gene was PCR-amplified from genomic DNA with primers HH32 (5′-GTGATGTTTTCGGTACAGCGGCCA-3′) and HH33 (5′-CTTTTATTCTGTGTGGAGATGTTC-3′), and subcloned into a PCR blunt II Topo vector (Invitrogen, http://www.invitrogen.com), to generate pROP18. The plasmid pROP18-Ty was designed to express a Ct Ty-tagged ROP18 protein in RH tachyzoites. It was constructed by inserting the coding sequence of ROP18 under the control of the tubulin promoter (TUB). The ROP18 gene coding sequence was PCR-amplified from pROP18 with forward primer HH51 (5′-ATG CAATTGATGTTT TCGGTACAGCGGCCA-3′; MfeI site underlined) and reverse primer HH50 (5′-TGCATGCATGTTCTGTGTGGAGATGTTCCTG-3′; NsiI site underlined), and subcloned as an MfeI/NsiI fragment into pTUB8mycGFPPftailTY, which was generously given by D. Soldati.

Plasmid pET-ROP18 was designed to express in E. coli a Ct His-tagged recombinant protein corresponding to the predicted kinase catalytic domain of ROP18. The DNA sequence coding for amino acids 243–539 was amplified by PCR from the pROP18 plasmid using forward primer HH40 (5′-GGGTTTCATATGACTACCGGTGAAACCCGG-3′; NdeI site underlined) and reverse primer HH41 (5′-AAATATGCGGCCGCTTCTGTGTGGAGATGTTC-3′; NotI site underlined) and cloning into NdeI and NotI sites of pET-24a vector (Novagen, http://www.emdbiosciences.com/html/NVG/home.html) to generate pET-ROP18.

Plasmid pTracer-ROP18ΔPro was designed to express the mature ROP18 protein spanning amino acids 83 to 539, such as the one stored in T. gondii rhoptries. It was constructed by PCR amplification of sequence from pROP18 plasmid using forward primer ML193 (5′-GCGGCCGCATGGAAAGGGCTCAACACCGGGTA-3′; NotI site underlined) and reverse primer ML194 (5′-TCTAGATTCTGTGTGGAGATGTTCCTG-3′; XbaI site underlined) and cloning into NotI and XbaI sites of pTracer-A 5 (Invitrogen). All constructs were verified by sequencing.

### Bioinformatic procedures.

PSI-BLAST program [26] was applied with standard parameters to search for homologous proteins in the Swiss-Prot Translated EMBL (SPTrEMBL) and National Center for Biotechnology Information (NCBI) non-redundant sequence databases. Fold-compatibility for the full-length and truncated sequences of ROP18 was searched and evaluated as previously described for other ROP2-like proteins [12]. Domain organisation was refined using fold-recognition results (see http://www.infobiosud.cnrs.fr/bioserver/ROP/suppl.html). Sequence–structure alignments, including ROP18 and its paralogs and distinct protein-kinases (see alignment in [12]), were manually refined with the help of the program ViTO [39]. Improved three-dimensional models were built for using MODELLER 7.0 with the loop optimization procedure.

SAXS data were collected from beamline X33 at Deutsches Elektronen-Synchrotron (DESY), European Molecular Biology Laboratory (EMBL) Hamburg. Data were collected at 10 °C, using a wavelength of λ = 1.5 Å. ROP2 was overexpressed and purified as previously described [30]. ROP2 was used at a concentration of 12.3 mg/ml in 0.2 M KPO4 (pH 8). Prior to data recording, the samples were extensively centrifuged to eliminate aggregates, and supplemented with 2 mM DTT. Diffusion spectra for buffer only were taken before and after the protein sample, averaged, and subtracted from the protein scattering curve. Data analysis and ab initio shape calculations were performed using PRIMUS, GNOM, GASBOR, and DAMMIN [40].

### Production of a recombinant ROP18 catalytic domain and of a specific antiserum.

The His-ROP18 recombinant protein was expressed in Plys E. coli (Stratagene, http://www.stratagene.com) that had been induced at 37 °C for 2 h with 1 mM isopropyl-β-D-thiogalactopyranoside. The bacterial pellets were resuspended in lysis buffer (Tris-HCl 50 mM [pH 7.5], NaCl 50 mM, EDTA 0.1 mM, and Complex mixture protease inhibitor tablet [Roche Applied Science, http://www.roche-applied-science.com]) and cells were broken using a French press (Thermo Spectronic; Thermo Scientific, http://www.thermo.com) operated at 20,000 p.s.i., then centrifuged at 12000*g* for 15 min; the pellet was washed with buffer (Tris-HCl 50 mM [pH 7.5], NaCl 50 mM, 0.1% Triton, and EDTA 0.1 mM). The protein was extracted from bacterial inclusion bodies by denaturation-refolding. Denaturation was performed in 6 M guanidinium chloride followed by ultracentrifugation of 100,000*g* for 30 min at 4 °C. The protein solution was then brought to 4 M guanidinium chloride. Refolding was obtained by a 10-fold dilution of the supernatant in the refolding buffer (Tris-HCl 50 mM [pH 8.3] and CsCl 100 mM). Proper refolding was assessed by DLS using a Zetasizer NanoZS (Malvern, http://www.malvern.com). The refolded His-ROP18 fusion protein was purified on Ni-NTA resin (Qiagen, http://www.qiagen.com) and eluted at 100 mM imidazole. The His-ROP18 D^394^A recombinant protein (see below) was expressed and refolded by the same procedure as the His-ROP18 recombinant protein.

Specific antibodies were obtained by subcutaneous immunizations of a rabbit with 1 mg of the recombinant protein separated on sodium dodecyl sulfate polyacrylamide gel electrophoresis (SDS-PAGE) and transferred to nitrocellulose, which was then crushed in Freund's complete adjuvant and injected. Two further injections were performed at 3-wk intervals in Freund's uncomplete adjuvant. The rabbit was then bled and the specific antibodies were affinity purified on the recombinant protein electrophoresed and Western blotted on nitrocellulose.

### Site-directed mutagenesis.

QuickChange Site-Directed Mutagenesis system (Stratagene) was used to introduce point mutation in the catalytic domain of ROP18 gene. The reaction was performed according to the manufacturer's instructions. Plasmid pET-ROP18 was used as template for construction of plasmid pET-ROP18-D^394^A where the triplet encoding the catalytic aspartic acid D^394^ has been changed to encode an alanine. The mutated catalytic domain sequence was obtained using primers HH54 (5′-ATTGTGCATACGGCTATCAAACCGGCG-3′) and HH59 (5′-CGCCGGTTTGATAGCCGTATGCACAAT-3′). The same mutation was also introduced in plasmid pROP18-Ty using the same primers to generate plasmid pROP18-Ty D^394^A, further identified as pROP18-TyMUT. The presence of the expected mutations was verified by sequencing.

### Parasite transfection and selection.

Transgenic parasites expressing ROP18-Ty or ROP18-TyMUT were obtained by electroporation of either 30 μg of pROP18-Ty or pROP18-TyMUT into 10^7^ HX tachyzoites as described previously [41]. After overnight growth, transfectants were selected with 25 μg/ml mycophenolic acid and 50 μg/ml xanthine, and cloned by limiting dilution under drug selection. Two independent transformation experiments were performed.

### BHK cells transient transfection experiments.

Transfections were carried out using Lipofectamine Reagent (GibcoBRL) as instructed by the manufacturer with 3 × 10^5^ BHK-21 cells grown on coverslips for 24 h in 6-well plates. After four h with Lipofectamine, cells were washed and incubated for four additional hours with complete BHK-21 medium. Then, the wells were infected with one million parasites for 16 h prior to fixation and immunofluorescence analysis.

### IFA.

For IFAs on intracellular parasites, HFFs were seeded on coverslips and infected with tachyzoites 24 h before fixation. Infected cells were fixed with 4% paraformaldehyde in phosphate buffered saline (PBS) for 30 min at room temperature, washed and permeabilized with 0.1% Triton X-100 in PBS for 10 min, and blocked with 10% FCS in PBS for 10 min. Coverslips were subsequently washed in PBS, then incubated with primary antibodies for 30 min at room temperature. Dilutions were 1:200 for mAb anti-V5, 1:100 for mAb anti-Ty and mAb T_3_ 4A_7_, 1:500 for rabbit anti-ROP1, and 1:10 for rabbit anti-ROP18 affinity-purified antibodies. Coverslips were washed in PBS and then incubated with affinity-purified goat anti-mouse immunoglobulin G (IgG) conjugated to FITC (Sigma, http://www.sigmaaldrich.com) and with goat anti-rabbit IgG conjugated to RITC (Jackson ImmunoResearch, http://www.jacksonimmuno.com) at 1:500 for 30 min. Finally, coverslips were washed and mounted onto microscope slides using Immunomount (Calbiochem, http://www.emdbiosciences.com/html/CBC/home.html).

IFA of invading parasites were done as described previously [8]. Briefly, after 2 min of invasion at 37 °C, coverslips were fixed with 4% paraformaldehyde in PBS and infected cells were permeabilized with 0.05% saponin in PBS. IFA was performed as described above. Cyt-D treatment was by incubating with 1 μM of the drug before and during invasion as described previously [18].

All observations were performed on a Leica DMRA2 microscope (Leica Microsystems, http://www.leica-microsystems.com) equipped for epifluorescence; images were recorded with a CoolSNAP CCD camera (Photometrics, http://www.photomet.com) driven by Metaview (Universal Imaging, http://www.moleculardevices.com) and processed using Adobe Photoshop 7.0 (Adobe Systems, http://www.adobe.com).

### SDS-PAGE and Western blotting.

Freshly released tachyzoites were boiled in SDS-PAGE sample buffer and separated on 10% polyacrylamide gels according to Laemmli [42]. M_r_ markers (Bio-Rad, http://www.bio-rad.com) were used for calibration. Proteins were transferred to nitrocellulose membranes (Protran; Schleicher & Schuell) at 0.8 mA/cm^2^ for 90 min by semi-dry transfer. The nitrocellulose strips were saturated for 1 h in 5% non-fat dry milk in 15 mM Tris-HCl (pH 8), 150 mM NaCl, and 0.05% Tween 20 (TNT). They were then incubated with mAbs (mouse ascitic fluids) or with polyclonal rabbit antibodies diluted 1:500 in TNT for 1 h. After washing, the strips were incubated with alkaline phosphatase–conjugated anti-mouse diluted 1:1000 in TNT and stained with BCIP-NBT.

### Immunosorption procedure.

Infected monolayers were solubilized in lysis buffer (Tris-HCl 50 mM [pH 8.3]/ NaCl 150 mM/ EDTA 4 mM/ PMSF 1 mM/1% Nonidet 40 -NP40-) for 1 h at 4 °C. The lysate was centrifuged 1 h at 16,000*g,* and the supernatant was collected for immunosorption. The immunosorbents were prepared by incubating 20 μl of ascitic fluid with 20 μl of Protein G-Sepharose for 1 h in 1 ml of PBS; they were then incubated with radiolabelled lysate at 4° C for 2 h under gentle agitation, washed four times with a buffer containing 1M NaCl and 0.5% NP40 in 50 mM Tris-HCl (pH 8.3) and then in 5mM Tris-HCl (pH 6.8). Elution was then performed during 5 min at 95 °C with electrophoresis sample buffer. After SDS-PAGE, the gel was impregnated with Amplify (Amersham, http://www.amershambiosciences.com), dried, and exposed to Biomax film (Kodak, http://www.kodak.com) at −80°C.

### Metabolic labeling and pulse-chase analysis.

Heavily infected HFF monolayers were incubated in methionine and cysteine-free DMEM (Invitrogen) containing 1% dialyzed FCS for 30 min at 37 °C in a 5% CO2 incubator prior to the addition of 50 μCi/ml [^35^S] methionine/cysteine (700 Ci/mM; MP Biomedicals, http://www.mpbio.com). The infected monolayers were then labeled for 30 min, rinsed with complete DMEM containing 10% FCS, and either arrested or incubated in this medium for 2 h chase prior to immunoprecipitation as described above.

### Kinase assays.

The assays were performed in a standard reaction buffer (30 μl) containing 25 mM Tris-HCl (pH 7.5), 15 mM MgCl_2_, 2 mM MnCl_2_, 15 μM ATP, and 20 μCi of [γ-^32^P]ATP (4500 Ci/mM; MP Biomedicals and Qbiogene, http://www.qbiogene.com) and the lysate of 10^7^ parasites or of 10^5^ HFFs that had been heated at 56 °C for 30 min to inactivate endogenous kinases. The reactions were initiated by addition of 10 μg each of the recombinant protein-kinase or of the recombinant mutated protein-kinase. The reaction proceeded for 30 min at 30 °C and was stopped by adding gel loading buffer and heating immediately at 95 °C for 5 min. Proteins were analyzed by electrophoresis on 12% SDS-polyacrylamide gel. The gels were dried and submitted to autoradiography.

## Supporting Information

Figure S1 Confocal Z Series through a BHK Cell That Has Been Transfected to Express a V5-Tagged ROP18Δpro and Infected Later by T. gondii (Arrow) GFP is expressed in the cytosol, whereas the V5 epitope is detected essentially at the PVM, but also between the parasites, inside the parasitophorous vacuole, at a location that may correspond to the PVM-derived tubulo-vesicular network.
(343 KB PPT)

Figure S2 SAXS Scattering Curve Obtained on ROP2, after Subtraction of the Buffer Contribution (Red Dots) The scattering curve calculated from the homology model is superimposed (blue line, χ^2^=15.6).
(41 KB PPT)

Figure S3 P(r) Distance Distribution of ROP2, Indicating a Maximum Particle Diameter of 13.5 nm, and a Structure Reminiscent of an Ellipsoid (White Arrow) with an Unstructured Extension (Black Arrow) (39 KB PPT)

Figure S4 Fit of a Homology Model of ROP2 into the Ab Initio SAXS Envelope The cartoon presentation of the homology model (magenta) was fitted into the best GASBOR pseudo-residue reconstruction (green spheres) obtained from ten individual trails (χ^2^ for best GASBOR model to data was 6.2). Left and right panels are perpendicular views.
(533 KB PPT)

Figure S5 DLS Spectra of Recombinant ROP2 (Red) and Refolded Recombinant ROP18 (Green) Observed sizes are similar (∼70 Å) and in agreement with those deduced from SAXS experiment performed on ROP2.
(64 KB PPT)

Figure S6 Intracellular Proliferation Rate at 16 h Post-Invasion by Wild-Type Tachyzoites (HX) and Tachyzoites Expressing an Additional Copy of ROP18-Ty (ROP18Ty) or a D^394^A-Mutated Version Thereof (ROP18Ty MUT) Graph representation of the mean number of parasites per vacuole (HX versus ROP18Ty: 5 experiments; HX versus ROP18TyMUT: 3 experiments).
(41 KB PPT)

### Accession Numbers

The GenBank (http://www.ncbi.nlm.nih.gov/Genbank) accession numbers for the sequences discussed in this paper are ROP2 (CAA85377), ROP4 (CAA96467), ROP5 (DQ116423), ROP7 (AM056071), ROP18 (AM075204), TgSUB2 (AF420596).

## Abbreviations

Ct: C-terminal
Cyt-D: cytochalasin-D
DLS: dynamic light scattering
FCS: fetal calf serum
GFP: green fluorescent protein
HFF: human foreskin fibroblast
HX: *T. gondii* strain RHΔhx
IFA: immunofluorescence assay
mAb: monoclonal antibody
Nt: N-terminal
PBS: phosphate buffered saline
PVM: parasitophorous vacuole membrane
SAXS: small angle X-ray scattering
SDS-PAGE: sodium dodecyl sulfate polyacrylamide gel electrophoresis
